# Depression, anxiety and coping mechanisms among mental healthcare practitioners during COVID-19

**DOI:** 10.4102/sajpsychiatry.v30i0.2307

**Published:** 2024-10-09

**Authors:** Yolandi Stals, Edwin du Plessis, Paul J. Pretorius, Mariette Nel, Alexander Boateng

**Affiliations:** 1 Department of Psychiatry, Faculty of Health Sciences, University of the Free State, Bloemfontein, South Africa; 2 Department of Psychology, Faculty of Humanities, University of the Free State, Bloemfontein, South Africa; 3 Department of Biostatistics, Faculty of Health Sciences, University of the Free State, Bloemfontein, South Africa

**Keywords:** COVID-19, depression, anxiety, mental healthcare, coping, coping strategies, mental healthcare practitioners, stress

## Abstract

**Background:**

The coronavirus disease 2019 (COVID-19) pandemic has placed the psychological functioning of mental healthcare practitioners under severe strain. Coping methods may affect mental health outcomes.

**Aim:**

The study examined the relationship between depression, anxiety, stress, and coping styles utilised by mental healthcare practitioners during the COVID-19 pandemic.

**Setting:**

Three private and one public mental healthcare facility in Bloemfontein, South Africa.

**Methods:**

Respondents completed the Depression, Anxiety and Stress Scale (DASS-21) and Brief Coping Orientation to Problems Experienced (Brief-COPE) inventories. An ordinal regression model was used to assess the relationship between coping styles, anxiety and depression.

**Results:**

A total of 212 practitioners were included in the analysis. According to DASS-21 measures, approximately 41% and 28% of respondents had moderate to severe depressive and anxiety symptoms, respectively, with the highest prevalence among younger female respondents and nurses. The association between stress severity, anxiety and depression was significant. Avoidant coping methods and two approach coping strategies (planning and acceptance) were associated with depression and anxiety. Anxiety was linked to an increased likelihood of transitioning to higher avoidant categories, while participants with depression were less likely to move to higher avoidant or approach categories.

**Conclusion:**

Mental healthcare practitioners, especially nurses, experienced significant COVID-19-related psychological distress during the pandemic. Avoidant coping mechanisms may increase the risk of poor mental health outcomes.

**Contribution:**

This study added data on the mental health effects of COVID-19 on mental healthcare practitioners, as well as psychological methods used to cope during the pandemic.

## Introduction

The coronavirus disease 2019 (COVID-19) pandemic caused high levels of psychosocial stress and posed significant challenges in providing healthcare, including in South Africa.^1,2^ The risk of acute and longstanding mental health consequences among healthcare practitioners resulting from a pandemic may compromise work performance and increase the risk of clinical error.^2^

Significant anxiety and distress have been reported in up to 36% of adults in the general population during the pandemic.^3^ Clinically significant psychological stress is more likely to occur in healthcare practitioners exposed to the COVID-19 virus, with high rates of depression (50.4%), anxiety (44.6%), insomnia (34%) and distress (71.5%) reported among healthcare practitioners during the pandemic.^3^ Although previous studies have reported on psychological distress and coping behaviours in the general population, students and healthcare practitioners during the pandemic, very few explored responses of those working in mental healthcare settings.^3,4,5,6,7,8,9,10,11^ Managing highly infectious diseases does not fall within mental healthcare practitioners’ normal scope of practice. Furthermore, before the availability of vaccines, preventative measures were often not practical in mental healthcare settings.^12^ Evidence suggests that mental healthcare practitioners exposed to poor work conditions, poorer patient outcomes, high stress levels and emotional exhaustion during the COVID-19 pandemic resulted in an increased risk of burnout.^13,14^ In addition, the prevalence of psychiatric symptoms during the pandemic exceeded baseline prevalence.^15,16^

The COVID-19 presents a range of neuropsychiatric disorders during the acute and post-illness phases. Confusion, depressed mood, anxiety, impaired memory and insomnia, and steroid-induced mania and psychosis are encountered during the acute stages of COVID-19.^15^ A recent meta-analysis indicated a high prevalence of post-traumatic stress disorder (PTSD) (32.2%), depression (14.9%) and anxiety disorders (14.8%) during the post-illness phase.^15^ An increased risk of incident anxiety, depressive stress, and adjustment and substance use disorders have also been reported at 1-year follow-up.^16^

The increase in neuropsychiatric complications has led to an influx of psychiatric patients to the mental healthcare system, which may impact the coping abilities of mental healthcare practitioners.^11^ More people have needed mental health support since the pandemic, but disruptions and resource shortages have aggravated the mental healthcare system’s ability to administer care.^17^ According to the World Health Organization,^17^ more than 60% of countries, including South Africa, reported disruptions in the delivery of mental health services. These included difficulties with delivering counselling and psychotherapy (67%), critical harm reduction (65%) and emergency interventions (35%).^17^

In an online survey on mental health conducted by the South African Depression and Anxiety Group (SADAG), more than half of the 1214 respondents reported anxiety and panic as one of the main challenges during the lockdown and has since recorded an increase in the number of calls to their helplines.^18^

**FIGURE 1:**
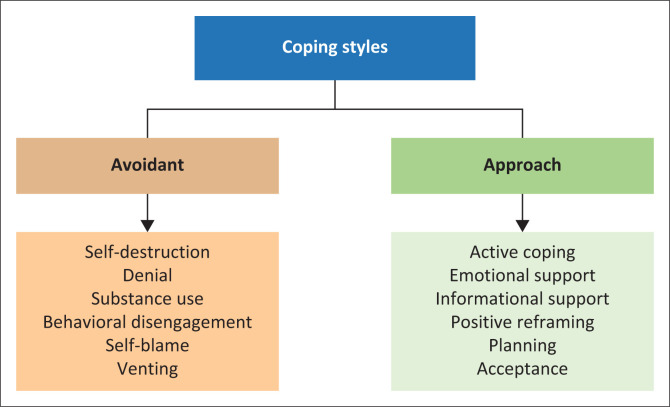
Brief-Coping Orientation to Problems Experienced inventory: Avoidant and approach coping styles.

Psychosocial responses, including coping strategies, are crucial in limiting adverse mental health outcomes in mental healthcare practitioners.^1,9^ Various coping strategies can be adopted to reduce psychological distress, with some variable efficacy, depending on the specific context.^19^ Contemporary coping mechanism analysis emphasises an avoidance and approach paradigm.^20^ Individuals cope with a stressor by avoiding it or attempting to adapt to or change it.^20^ Avoidant coping methods are considered dysfunctional and maladaptive and increase the risk of anxiety and depression.^20,21^ Approach coping strategies that include emotion- and problem-focused strategies are regarded as more adaptive and associated with better mental health outcomes.^20,21,22,23,24,25^ Avoidant or dysfunctional strategies include substance misuse, self-distraction, behavioural disengagement, denial, self-blame and venting^20^ (Figure 1).

Approach coping styles include problem- and emotion-focused strategies and, in most studies, are associated with better mental health and lower anxiety.^22,23,24,25^ Approach methods include active coping, planning, instrumental support (problem-focused), emotional support, positive reframing and acceptance. However, planning has previously been linked to higher psychological distress.^26^ The original Brief Coping Orientation to Problems Experienced (Brief-COPE) questionnaire included humour and religion as general coping factors. Later, their position became more ambiguous, with factor analysis indicating varying positions between approach and avoidant strategies.^27^

Coping strategies associated with better mental health outcomes during the recent COVID-19 pandemic are slowly emerging. Research from previous infectious disease outbreaks and the recent COVID-19 pandemic suggests that positive emotion-focused coping strategies, including positive reframing, humour and acceptance, can mitigate the effects of stress during a pandemic.^7,26,28^ The increased resilience with religion to various stressors reported in earlier studies was not replicated in recent studies.^26,29,30^ Other researchers reported that approach-coping methods, including problem-solving, cognitive restructuring, and problem-focused approach-coping strategies, such as planning and active coping, improved mental health during epidemics.^31,32,33,34^

The objectives of the study were: (1) to explore possible relationships between depression, anxiety and coping styles among mental healthcare practitioners during the COVID-19 pandemic; (2) to measure the severity of depression, anxiety and stress; and (3) to identify avoidant and approach coping methods used.

## Research methods and design

### Study design, population and sampling

A quantitative, descriptive, cross-sectional study was conducted. All mental healthcare practitioners working at Optima, Bloemcare and Mondia Woodlands private hospitals and the Free State Psychiatric Complex (FSPC) public healthcare facility were invited to participate. Professional categories among those invited included psychiatrists, psychologists, occupational therapists, social workers and nursing staff. The estimated population size was 740, of which 506 were employed in the public sector and 234 in the private sector.

### Data collection

The first author collected data from 01 August until 30 September 2021 during adjusted COVID-19 alert levels 3 and 2.^35^ A demographic questionnaire was designed to collect information regarding occupation, gender, age and years of clinical experience. In addition, the Depression, Anxiety and Stress Scale (DASS-21)^36^ and the Brief-COPE^37^ inventory were completed by each respondent.

### Depression, Anxiety and Stress Scale-21

The DASS-21^36^ measures psychological distress. Items on this scale describe negative emotional states experienced during the previous 7 days. It consists of 21 questions, with each scored on a four-point Likert scale from 0 (‘did not apply to me at all’) to 3 (‘applied to me very much’). Depression, anxiety and stress are measured by seven questions each. Depression, anxiety and stress were graded as normal, mild, moderate, severe or extremely severe during the calculation of participants’ Likert scores mentioned above (Table 2). The DASS-21 has previously been used in research on the COVID-19 pandemic.^7,38^ In a study conducted in Nigeria, Coker et al.^39^ reported excellent reliability for the DASS-21 subscales of depression, anxiety and stress (Cronbach’s alpha values of 0.81, 0.89 and 0.78, respectively). Furthermore, excellent internal consistency and discriminative, concurrent and convergent validities were demonstrated.^39^

For this study, respondents with normal or mild depression DASS-21 scores (≤ 13) and anxiety DASS-21 scores (≤ 9) were regarded as having low levels of depression and anxiety. Respondents with moderate, severe or extremely severe DASS-21 scores for depression (≥ 14) or anxiety (≥ 10) were regarded as having high levels of depression and anxiety.

### Brief-Coping Orientation to Problems Experienced questionnaire

For this study, the Brief-COPE^37^ was used to measure coping methods utilised by respondents. Avoidant or approach coping styles were compared for depression and anxiety. The Brief-COPE^37^ is a frequently used 28-item self-report questionnaire assessing theoretically derived coping mechanisms with 14 subscales. Each subscale consists of two items that are rated on a four-point Likert scale indicating 1 (‘I have not been doing this at all’), 2 (‘a little bit’), 3 (‘medium amount’) to 4 (‘I have been doing this a lot’).^9^ The scale can also determine someone’s primary coping styles as avoidant or approach coping (Figure 1).

The Brief-COPE has been used to evaluate coping strategies during previous infectious disease outbreaks. Hanfstingl et al.^40^ conducted a study during the COVID-19 pandemic on 529 respondents, of which the results supported the original hypothesised 14-factor structure of the Brief-COPE.

### Avoidant and approach coping styles

Avoidance strategies are cognitive, emotional or behavioural attempts to detach oneself from potentially harmful situations.^38^ These include efforts to deny, disengage emotionally or remove oneself physically from stressful situations. Brief-COPE avoidant coping domains include substance use, behavioural disengagement, self-distraction, self-blame, denial and venting (Figure 1). With approach coping strategies, individuals actively move towards stressors to alter the situation or seek alternatives.^38^ Strategies may include seeking information, social support and planning. On the Brief-COPE questionnaire, approach strategies include active coping, positive reframing, planning, acceptance, emotional and informational support. Religion and humour were excluded from the approach or avoidant paradigm because of their ambiguous position, as reported by previous investigators with factor analysis indicating varying positions between approach and avoidant strategies.^27^

### Data analysis

The Department of Biostatistics, Faculty of Health Sciences, University of the Free State analysed the data using SAS Version 9.4 and SPSS IBM Statistical software (SAS Institute Inc., Cary, NC, USA). Categorical variables were summarised as medians and percentiles as the distributions were skewed. Associations were calculated between health practitioners reporting low and high levels of depression and anxiety using Kruskal-Wallis for numerical variables and Chi-square or Fisher’s exact test for categorical variables.

Depression and anxiety were each compared with the variables of coping and stress. To evaluate the relationship between depression, anxiety and coping styles, we used an ordinal regression model that considered the hierarchical nature of coping strategies and how they might influence mental health outcomes. This model is particularly useful because it captures the hierarchy in coping strategies, analyses multiple mental health outcomes, provides increased statistical power, and permits context-specific analysis.

### Ethical considerations

Ethical clearance to conduct this study was obtained from the University of the Free State Health Sciences Research Ethics Committee (No. UFS-HSD2021/0039/2004). All ethical standards as prescribed by the Declaration of Helsinki were adhered to. Permission to conduct the study was obtained from the clinical managers of each hospital and the Free State Province Department of Health. Once approval was obtained, the first author distributed the information leaflets, informed consent documents and questionnaires to individual practitioners and supervisors for distribution. Participants placed completed questionnaires in strategically placed collection boxes in each facility.

Information remained confidential, and data were anonymised. Respondents placed completed questionnaires in a sealed collection box made available at each facility, collected by the first author. Respondents were provided with the principal investigator’s contact number. They were encouraged to contact the investigator or another mental healthcare practitioner of their choice if they experienced severe anxiety or depression during or after completing the questionnaire.

## Results

### Demographic data

A total of 212 (28.6%) of 740 mental healthcare practitioners in Bloemfontein’s private and public mental health sectors participated in the study (Table 1). The majority were women (81.1%), nursing staff (77.8%) and between the ages of 46–55 years (29.2%), followed by 36–45 years (26.4%) and 26–35 years (24.1%). Most respondents worked in the public sector (60.4%), followed by the private sector (28.3%), and had between 0 and 5 years (27.8%) or 5–10 years of experience (27.4%).

**TABLE 1:** Demographic characteristics of respondents with high depression and anxiety scores.

Demographic characteristics	Total group (*N* = 212)		Depression*		Anxiety**	
			Score ≥ 14		Score ≥ 10	
	*n*	%	*n*	%	*n*	%
**Gender**						
Male	40	19.0	8	20.0	13	32.0
Female	172	81.0	52	30.0	73	42.0
**Age range (years)**						
18–25	10	5.0	4	40.0	8	80.0
26–35	51	24.0	20	39.0	25	49.0
36–45	56	26.0	14	25.0	21	37.0
46–55	62	29.0	15	24.0	19	31.0
56–65	27	13.0	5	18.0	9	33.0
> 65	6	3.0	2	33.0	4	67.0
**Occupation**						
Nursing	165	78.0	52	31.0	73	44.0
Social worker	3	1.0	1	33.0	1	33.0
Occupational therapist	15	7.0	2	13.0	5	33.0
Psychologist	16	7.0	4	25.0	6	37.0
Psychiatrist/medical practitioner	13	6.0	1	8.0	1	8.0
**Years of experience (years)**						
0–5	59	28.0	23	39.0	32	54.0
5–10	58	27.0	16	28.0	24	41.0
10–20	32	15.0	5	16.0	10	31.0
20–30	32	15.0	8	25.0	9	28.0
30–40	23	11.0	6	26.0	7	30.0
> 40	8	4.0	2	25.0	4	50.0
**Workplace**						
Private sector	60	28.0	19	32.0	24	40.0
Public sector	128	60.0	34	27.0	51	40.0
Both private and public sector	24	11.0	7	29.0	11	46.0

* , DASS-21 depression scores: 0–13 = normal-mild; ≥ 14 = moderate, severe and extremely severe.

** , DASS-21 anxiety scores: 0–9 = normal-mild, ≥ 10 = moderate, severe, and extremely severe.

### Depression and anxiety

Overall, 28.3% and 40.6% of respondents, primarily women, reported high levels of depression and anxiety, respectively (Table 1). Professional categories with high depression scores (≥ 14 on the DASS-21) mainly included nurses (31.5%). Depression, Anxiety and Stress Scale-21 anxiety scores ≥ 10 (moderate to extremely severe) were also high for 44.2% of nurses, followed by psychologists (37.5%). Only 7.7% of medical practitioners reported moderate or severe depression and anxiety.

### Depression, anxiety and stress

A significant association (*p* < 0.0001) between depression, anxiety and stress severity was observed in our study population (Table 2). Respondents with high levels of depression and anxiety experienced high stress levels in 63.3% and 55.9% of cases, respectively. Most respondents (80.3%) with low DASS-21 depression scores (≤ 13) had normal stress levels (DASS-21 stress scores ≤ 14), while 10.5% and 9.2% regarded their stress as mild, or moderate to severe, respectively. Normal to mild anxiety was associated with normal (86.5%), mild (10.3%) or moderate (3.2%) stress exposure.

**TABLE 2:** Depression, Anxiety and Stress Scale-21 depression and anxiety scores by stress severity.

Distribution of scores	DASS-21 stress severity scores										*p*
	Normal (0–14)		Mild (15–18)		Moderate (19–25)		Severe (26–33)		Extremely severe (34–42)		
	*n*	%	*n*	%	*n*	%	*n*	%	*n*	%	
Depression*											< 0.0001#
≤ 13 (*n* = 152)	122	80.3	16	10.0	12	8.0	2	1.0	0	0.0	
≥ 14 (*n* = 60)	9	15.0	13	22.0	17	28.0	12	20.0	9	15.0	
Anxiety**											< 0.0001#
≤ 9 (*n* = 126)	109	86.0	13	10.0	4	3.2	0	0.0	0	0.0	
≥ 10 (*n* = 86)	22	26.0	16	19.0	25	29.0	14	16.0	9	10.0	

DASS, Depression, Anxiety and Stress Scale.

# , Fisher’s exact test was used to determine the *p*-value because of small expected counts.

* , DASS-21 depression scores: 0–13 = normal-mild, ≥ 14 = moderate, severe and extremely severe;

** , DASS-21 anxiety scores: 0–9 = normal-mild, ≥ 10 = moderate, severe, and extremely severe.

### Avoidant versus approach coping styles in depression and anxiety

Avoidant coping strategies predicted both depression (*p* < 0.0001) and anxiety (*p* < 0.0001), while approach coping strategies were associated with anxiety only (*p* = 0.0068) (Table 3). Respondents with high depression or anxiety scores used avoidant coping strategies (‘little bit, medium, a lot’) in 91.7% and 82.6% of cases, respectively (*p* < 0.0001) (Table 4). Most respondents with low levels of depression (62.5%) or anxiety (67.5%) did not use avoidant coping. Approximately 57% of those with high anxiety levels used approach coping (47.7% ‘medium’, 9.3% ‘a lot’), while 48.5% of respondents with low anxiety used approach coping moderately or frequently (31.8% ‘medium’, 16.7% ‘a lot’) (*p* = 0.0068) (Table 5).

**TABLE 3:** Depression, Anxiety and Stress Scale-21 depression and anxiety scores by avoidant and approach coping style.

DASS-21 scores	Coping style								*p*
	Not at all		Little bit		Medium amount		A lot		
	*n*	%	*n*	%	*n*	%	*n*	%	
**Avoidant coping style**									
Depression*									< 0.0001#
≤ 13 (*n* = 152)	95	62.0	48	32.0	9	6.0	0	0.0	
≥ 14 (*n* = 60)	5	8.0	42	78.0	11	18.0	1	2.0	
Anxiety**									< 0.0001#
≤ 9 (*n* = 126)	85	67.0	34	27.0	7	6.0	0	0.0	
≥ 10 (*n* = 86)	15	17.0	57	66.3	13	15.0	1	1.0	
Depression*									0.1195
≤ 13 (*n* = 152)	39	26.0	36	24.0	54	35.0	23	15.0	
≥ 14 (*n* = 60)	8	13.0	19	32.0	27	45.0	6	10.0	
Anxiety**									0.0068
≤ 9 (*n* = 126)	36	29.0	29	23.0	40	32.0	21	17.0	
≥ 10 (*n* = 86)	11	13.0	26	30.0	41	48.0	8	9.0	

DASS, Depression, Anxiety and Stress Scale.

# , Fisher’s exact test was used to determine the *p*-value because of small expected counts.

* , DASS-21 depression scores: 0–13 = normal-mild, ≥ 14 = moderate, severe and extremely severe.

** , DASS-21 anxiety scores: 0–9 = normal-mild, ≥ 10 = moderate, severe and extremely severe.

**TABLE 4:** Avoidant coping in depression and anxiety.

Types of avoidant coping	DASS-21 depression scores					DASS-21 anxiety scores				
	Normal-mild (≤ 13)		Moderate-extremely severe (≥ 14)		*p*	Normal-mild (≤ 9)		Moderate-extremely severe (≥ 10)		*p*
	*N* = 152		*N* = 60			*N* = 126		*N* = 86		
	*n*	%	*n*	%		*n*	%	*n*	%	
**Self-distraction**					0.0031#					0.0003#
Not at all	48	32.0	5	8.0		45	36.8	9	10.0	
Little bit	57	37.0	42	72.0		47	37.0	37	43.0	
Medium amount	38	25.0	11	18.0		30	24.0	33	38.0	
A lot	9	6.0	1	2.0		4	3.0	7	8.0	
**Denial**					< 0.0001#					0.0002#
Not at all	115	76.0	23	38.0		97	77.0	41	48.0	
Little bit	24	16.0	20	33.0		18	14.0	26	30.0	
Medium amount	9	6.0	12	20.0		8	6.0	13	15.0	
A lot	4	3.0	5	8.0		3	2.0	6	7.0	
**Substance use**					< 0.0001#					< 0.0001#
Not at all	127	84.0	33	55.0		107	85.0	53	62.0	
Little bit	20	13.0	15	25.0		15	12.0	20	23.0	
Medium amount	3	2.0	9	15.0		1	1.0	11	13.0	
A lot	2	1.32	3	5.0		3	2.0	2	2.0	
**Behavioural disengagement**					< 0.0001#					< 0.0001#
Not at all	123	81.0	26	43.0		104	82.0	45	52.0	
Little bit	25	16.0	22	37.0		20	16.0	27	31.0	
Medium amount	3	2.0	10	17.0		2	2.0	11	13.0	
A lot	1	1.0	2	3.0		0	0	3	3.0	
**Self-blame**					< 0.0001#					< 0.0001#
Not at all	102	67.0	14	23.0		89	71.0	27	31.0	
Little bit	39	26.0	19	32.0		28	22.0	30	35.0	
Medium amount	10	7.0	20	33.0		8	6.0	22	26.0	
A lot	1	1.0	7	12.0		1	1.0	7	8.0	
**Venting**					0.0054#					0.0007#
Not at all	70	46.0	13	22.0		63	50.0	20	23.0	
Little bit	54	35.0	34	57.0		42	33.0	46	53.0	
Medium amount	21	14.0	11	18.0		15	12.0	17	20.0	
A lot	7	5.0	2	3.3		6	5.0	3	3.0	

DASS-21, Depression, Anxiety and Stress Scale-21.

# , Fisher’s exact test was used to determine the *p*-value because of small expected counts.

**TABLE 5:** Approach coping with depression and anxiety.

Brief-COPE approach coping styles	DASS-21 depression scores					DASS-21 anxiety scores				
	Normal-mild (≤ 13)		Moderate-extremely severe (≥ 14)		*p*	Normal-mild (≤ 9)		Moderate-extremely severe (≥ 10)		*p*
	*N* = 152		*N* = 60			*N* = 126		*N* = 86		
	*n*	%	*n*	%		*n*	%	*n*	%	
**Active coping**					0.0553					0.0045*
Not at all	41	27.0	9	15.0		38	30.0	12	14.0	
Little bit	50	33.0	28	47.0		39	31.0	39	45.0	
Medium amount	42	28.0	20	33.0		32	25.0	30	35.0	
A lot	19	12.0	3	5.0		17	13.0	5	6.0	
**Emotional support**										
Not at all	44	29.0	12	20.0	0.1597	39	31.0	17	20.0	0.1630
Little bit	52	34.0	25	42.0		43	34.0	34	39.0	
Medium amount	32	21.0	18	30.0		25	20.0	25	29.0	
A lot	24	16.0	5	8.0		19	15.0	10	12.0	
**Informational support**										
Not at all	46	30.0	14	23.0	0.7748	43	34.0	17	20.0	0.0321*
Little bit	53	35.0	24	40.0		43	34.0	34	39.0	
Medium amount	40	26.0	17	28.0		27	21.0	30	35.0	
A lot	13	7.0	5	8.0		13	10.0	5	6.0	
**Positive reframing**										
Not at all	40	26.0	10	17.0	0.0845	37	29.0	13	15.0	0.0677
Little bit	63	41.0	20	33.0		49	39.0	34	39.0	
Medium amount	34	22.0	23	38.0		28	22.0	29	34.0	
A lot	15	10.0	7	12.0		12	9.0	10	12.0	
**Planning**										
Not at all	47	31.0	10	17.0	0.0037*	44	34.9	13	15.0	0.0025*
Little bit	35	23.0	19	32.0		25	19.8	29	34.0	
Medium amount	44	29.0	28	47.0		37	29.4	35	41.0	
A lot	26	17.0	3	5.0		20	15.9	9	10.0	
**Acceptance**										
Not at all	40	26.0	6	10.0	0.0065*	37	29.0	9	10.0	0.0059*
Little bit	42	28.0	28	30.0		29	23.0	31	36.0	
Medium amount	44	29.0	30	50.0		40	32.0	34	39.0	
A lot	26	17.0	6	10.0		20	16.0	12	14.0	

DASS-21, Depression, Anxiety and Stress Scale-21; COPE, Coping Orientation to Problems Experienced.

* , Statistically significant.

### Selected avoidant coping methods: Depression and anxiety

Significant associations between depression, anxiety and all avoidant coping styles were identified in our study population (Table 6). Six avoidant coping strategies were used significantly more by depressed and anxious respondents independently. The associations were particularly robust for self-blame, behavioural disengagement and substance use (*p* < 0.0001). The other avoidant styles included denial, self-distraction and venting. Respondents with high depression scores used self-distraction (91.7%), self-blame (76.7%), denial (61.6%) and substance use (45%) more often as coping strategies. Most respondents (80.9%) with low depression scores did not disengage behaviourally (*p* < 0.0001) as a means of coping.

**TABLE 6:** Brief-Coping Orientation to Problems Experienced factorial structure: clinically significant associations for depression and anxiety.

Coping mechanisms	Depression (High vs. low) *p*-values	Anxiety (High vs. low) *p*-values
**Avoidant coping**	< 0.0001*	< 0.0001*
Self-distraction	0.0031*	0.0003*
Denial	< 0.0001*	0.0002*
Substance use	< 0.0001*	< 0.0001*
Behavioural disengagement	< 0.0001*	< 0.0001*
Self-blame	< 0.0001*	< 0.0001*
Venting	0.0007*	0.0054*
**Approach coping**	0.1195	0.0068*
Active coping	0.1195	0.0045*
Emotional support	0.1597	0.1630
Informational support	0.7748	0.0321*
Positive reframing	0.0845	0.0677
Planning	0.0037*	0.0025*
Acceptance	0.0065*	0.0059*

COPE, Coping Orientation to Problems Experienced.

* , Statistically significant.

Similarly, those with high anxiety scores used denial (52.3%), self-blame (68.6%), substance use (38.4%) and venting (76.8%) significantly more than those with normal or mild anxiety. In contrast, significantly fewer with normal or mild anxiety used avoidance mechanisms such as denial (77.0% ‘a lot’), self-blame (70.6% ‘a lot’), substance use (84.9% ‘a lot’) or behavioural disengagement (82.5% ‘a lot’) as coping strategies.

### Approach coping subscales: Depression and anxiety

Of all the different approach coping styles investigated in this study, active coping (*p* = 0.0045), informational support (*p* = 0.0321), and planning (*p* = 0.0025) were associated with anxiety. Planning and acceptance were also associated with depression (*p* = 0.0037 and 0.0065, respectively) (Table 5). No significant differences in other approach coping strategies, including seeking emotional support, informational support and positive reframing, were found among respondents with and without depression or anxiety.

### Other coping methods: Anxiety and depression

Respondents with high anxiety levels used humour and religion more (65.5% and 88.4%, respectively) than those with low anxiety (46.8% and 75.4%, respectively) (*p* = 0.0087 and 0.0188, respectively). Respondents with high depression scores used humour more (66.7%) than those with low depression scores (49.3%) (*p* = 0.0226). Using religion to cope was similar across the range of DASS-21 depression scores. Respondents who did not use humour or religion for coping reported significantly less anxiety (*p* < 0.0001) and depression (*p* < 0.0001). The differences remained significant for primarily avoidant or approach coping styles (Table 7).

**TABLE 7:** Depression, Anxiety and Stress Scale-21 scores, religion and humour.

DASS-21 scores	No humour						Humour						*p*
	No religion (*n* = 29)			Religion (*n* = 12)			No religion (*n* = 68)			Religion (*n* = 103)			
	Median	IQR	Range	Median	IQR	Range	Median	IQR	Range	Median	IQR	Range	
Depression*	0	0–2.0	0–42.0	15.0	8.0–20.0	2.0–40.0	8.0	2.0–14.0	0–42.0	8.0	4.0–16.0	0–40.0	< 0.0001#
Anxiety**	0	0–2.0	0–38.0	12.0	5.0–17.0	0–40.0	6.0	2.0–14.0	0–38.0	8.0	4.0–16.0	0–34.0	< 0.0001#

IQR, interquartile range; DASS-21, Depression, Anxiety and Stress Scale-21.

* , DASS-21 depression scores: 0–13 = normal-mild, ≥ 14 = moderate, severe and extremely severe;

** , DASS-21 anxiety scores: 0–9 = normal-mild, ≥ 10 = moderate, severe and extremely severe.

# , Statistically significant.

## Discussion

The study demonstrated satisfactory reliability and validity for the DASS-21 (α = 0.95) and the Brief-COPE (α = 0.92). This study replicated previous findings of a relationship between depression or anxiety and stress.^41^ In the context of personal protective equipment (PPE) shortages, fear of contracting COVID-19, a high workload, being confronted by moral and difficult ethical decisions, and reliance on effective coping strategies became paramount for limiting mental distress among medical personnel during the pandemic.^42^ Practitioners in mental healthcare settings are particularly vulnerable as they are expected to function outside their usual scope of practice. Furthermore, managing highly infectious diseases, often in crowded wards where the implementation of social distancing and other preventative measures are unfeasible, may significantly contribute to stress among mental healthcare practitioners.^43^

Mental healthcare practitioners in Bloemfontein demonstrated a range of psychological responses to the COVID-19 outbreak. Salari et al.^42^ reported high prevalence rates for depression (33.7%) and anxiety (31.9%) in the general population during the COVID-19 pandemic. Similar prevalence rates were reported among healthcare practitioners, ranging between 25.8% and 28.3% for depression and 24.9% and 36% for anxiety.^44,45^ Respondents in this study reported comparable rates of depression (28.3%). However, the prevalence of anxiety exceeded those reported in previous similar studies (40.6%).^42,44,45^

In this study, female gender, age 26–35 years and fewer years of professional experience were associated with depression and anxiety. These findings confirm previous reports that identified female gender, younger age and less professional experience as independent risk factors for depression and anxiety.^3,46^ A lack of experience, fear of expressing challenges, higher error rates, and higher incidences of unhealthy behaviour may contribute towards the vulnerability of younger staff to the adverse mental effects of stress.^47^

Nurses (77.8%) were the largest participating professional group in this study and presented the highest risk for depression and anxiety. Our finding confirms previous reports of nursing staff as the professional category most at risk of mental illness during a pandemic.^47,48^ Nurses are frontline workers who experience the direct impact when healthcare systems are strained, thus making them more vulnerable to depression, anxiety and stress.^48^ Previous research highlighted the association between high core competencies and a lower prevalence of psychological distress among healthcare professionals during COVID-19.^49^

These findings are supported by the second-highest rates of depression and anxiety reported by social workers and clinical psychologists in this study. Working directly with highly infectious patients does not form part of the core competencies of social workers or clinical psychologists. Psychiatrists and medical practitioners presented the lowest risk for depression and anxiety.

### Stress

Evidence suggests a critical role for stress in the structure of negative mood states, including depression and anxiety.^41^ Interventions mitigating the negative effects of stress may be particularly effective in reducing depression and anxiety symptoms.^41^ Coping strategies play a significant role in determining outcomes during exposure to stressful events and are important determinants of psychological well-being, functioning and the development of medical and psychiatric illnesses.^41,50^

In this study, a small percentage of respondents (10.9%) reported experiencing severe to extremely severe stress on the DASS-21. Most respondents (80.3%) with low depression scores reported normal stress. Similarly, 86.5% of respondents with low anxiety scores experienced normal stress. Although associations between stress and depression and stress and anxiety were demonstrated in the study population, the possible moderating role of effective coping strategies on stress is unclear.

### Avoidant coping

Similar to previous studies, avoidant coping styles correlated independently with higher DASS-21 depression and anxiety scores in our study population.^27,51,52^ In particular, substance use, behavioural disengagement, and self-blame were highly significant for both depression and anxiety. Avoidant coping strategies were never utilised, or to a limited extent, by respondents with low depression (94%) and anxiety (94.5%). This suggests that avoidant coping may be a risk factor for anxiety, as reported previously.^27,51^ Bistricky^51^ and Lopes et al.^52^ reported similar associations between depression, anxiety, behavioural disengagement, denial, self-blame, self-distraction and substance use during disasters.

### Approach coping

An intriguing finding in this study was that significantly more (*p* = 0.0068) respondents with high anxiety scores (57.0%) regularly used approach coping methods (active coping, *p* = 0.0045 and planning, *p* = 0.0025) compared to those with low anxiety scores (48.5%) (Table 6). Active coping and planning were the two approach coping factors that reached the highest statistical differences between those with low and high anxiety scores. An intolerance of uncertainty could explain the unexpectedly high rate of approach coping in this subgroup, although it will require further investigation. Hoffman et al.^53^ have argued that the level of anxiety determines the coping style, that is, those with low levels of anxiety do not require approach coping, which will support our previous statement. On the other hand, individuals who use approach coping may reduce anxiety through repeated exposure to anxiety-provoking stimuli that eventually result in extinction. Hesitant or inconsistent (intermediate) use of approach coping is, therefore, more likely to result in high levels of anxiety. It is also possible that the relative brevity of exposure during the pandemic could have been insufficient to result in extinction through exposure.

Planning and acceptance were significantly associated with depression and anxiety among those with approach coping styles. Both planning and active coping positively correlated with anxiety, while planning and acceptance correlated with depression. These findings contradict previous research that reported planning and acceptance coping with being protective against depression and anxiety.^7,26,28^ A recent study conducted on the general population by Miola et al.^54^ during COVID-19 found that planning predicted low depression. It is reasonable to accept that, in a mental healthcare setting, too many complex and unpredictable factors can frustrate planning attempts and may instead increase stress. Savitsky et al.^26^ reported findings similar to ours, where planning was associated with more psychological distress among nursing students during the COVID-19 pandemic. Planning may form part of excessive worry, rumination, online research, and grieving during the pandemic. It is also possible that respondents could not choose or plan a safe approach but were required to continue managing patients under potentially life-threatening circumstances with uncertain outcomes for themselves and the patients. Exposure to overwhelming illness, trauma, and loss in the face of limited resources could also result in coping through acceptance as part of the grief process.

### Humour and religion

Low levels of anxiety and depression correlated significantly with never using religion or humour as a means of coping. Evidence suggests that the beneficial effects of humour as a coping style depend on the perceived availability of social support. Using humour in the context of a perceived lack of social support or self-denigrating humour (maladaptive use) has been linked to depression and anxiety.^55^ Healthcare practitioners reported a significant lack of and need for social support during the pandemic, which may explain the possible maladaptive effects of humour in our study population.^56^

Respondents with low DASS-21 anxiety scores used religion significantly more (regularly to very regularly) than those with high scores. Those with high anxiety scores used religion ‘a little bit’ (38.4%), ‘medium’ (20.9%) or ‘a lot’ (29.1%).

### Further analysis

The study found that heightened anxiety was linked to an increased likelihood of transitioning to higher categories on the avoidant coping scale. Individuals with humour and religious attributes were less likely to transition to higher categories on the avoidant scale. Additionally, depression was associated with a reduced likelihood of moving to higher categories on both avoidant and approach coping scales. These findings highlight the complex interplay of emotional factors on coping strategies, emphasising the need for targeted interventions that address anxiety, depression, and psychosocial support among healthcare practitioners. Such interventions could enhance well-being, resilience, and coping strategies, ultimately contributing to the overall mental health of healthcare professionals on the frontlines of the pandemic response.

The cross-sectional study design may have limitations in predicting the long-term relationship between variables. Our research does not address the question of directionality or cause and effect. Do anxious or depressed mental healthcare practitioners engage in coping styles because they are anxious or depressed, or do specific coping mechanisms increase an individual’s risk of mental difficulties?

Although it may be possible that the low response rate could have affected study results, potential respondents who were already emotionally overburdened may have chosen not to respond. In addition, the challenges imposed on mental healthcare practitioners during the pandemic and concerns about confidentiality and anonymity could have influenced decisions about participation. The sample was drawn from a limited geographic location in South Africa and the results may not be generalisable to other centres.

## Conclusion

Analysing coping strategies among mental healthcare practitioners during the COVID-19 pandemic revealed significant associations between psychological states and coping mechanisms. Our study confirms the significant psychological effects of COVID-19 on mental healthcare practitioners, especially nursing staff. Women and younger staff are at risk for depression and anxiety. Mental healthcare practitioners must be able to identify avoidant coping strategies that may increase the risk for depression and anxiety. Previous research suggests a protective role for approaching coping methods, but that finding was not replicated in this study.
